# From theory to practice: improving the impact of health services research

**DOI:** 10.1186/1472-6963-5-1

**Published:** 2005-01-07

**Authors:** Kevin Brazil, Elizabeth Ozer, Michelle M Cloutier, Robert Levine, Daniel Stryer

**Affiliations:** 1 Department of Clinical Epidemiology and Biostatistics, Faculty of Health Sciences, McMaster University and St. Joseph's Health System Research Network, Hamilton, ON, Canada; 2 Department of Pediatrics/Adolescent Medicine, University of California, San Francisco, CA, USA; 3 Department of Pediatrics, University of Connecticut Health Center and Connecticut. Children's Medical Center, Hartford, CT, USA; 4 Occupational and Preventive Medicine, Meharry Medical College, Nashville, TN, USA; 5 Center for Outcomes and Effectiveness Research, Agency for Healthcare Research and Quality, Rockville, MD, USA

## Abstract

**Background:**

While significant strides have been made in health research, the incorporation of research evidence into healthcare decision-making has been marginal. The purpose of this paper is to provide an overview of how the utility of health services research can be improved through the use of theory. Integrating theory into health services research can improve research methodology and encourage stronger collaboration with decision-makers.

**Discussion:**

Recognizing the importance of theory calls for new expectations in the practice of health services research. These include: the formation of interdisciplinary research teams; broadening the training for those who will practice health services research; and supportive organizational conditions that promote collaboration between researchers and decision makers. Further, funding bodies can provide a significant role in guiding and supporting the use of theory in the practice of health services research.

**Summary:**

Institutions and researchers should incorporate the use of theory if health services research is to fulfill its potential for improving the delivery of health care.

## Background

While significant strides have been made in medical research over the past several decades, many research results considered important by researchers and expert committees are not being used by health care practitioners. While the value of health services research must be judged by its validity, its utility cannot be taken for granted. There has been an assumption that when research information is available it will be accessed, appraised and then applied [1]. However, knowledge of a research-based recommendation is by itself insufficient to ensure its adoption. While the value of research evidence as a basis for decision making in health care is well established, the incorporation of such evidence into decision-making remains inconsistent [2].

The gap between research evidence and its' incorporation into practice has led to an increase in research in how to bring new knowledge to bear on everyday health care. Factors influencing the adoption of research evidence have been studied extensively [3-5]. Personal attributes, time, organizational boundaries, geography and educational background all contribute to decision-makers' responses to research evidence [6,7]. An area that has received less attention is the incorporation of theory in health services research. The authors of this paper propose a need for a stronger theoretical base in health services research wherein health services research would be more informative and influential, facilitating the adoption of research results into practise. Integrating theory into health services research is an important first step. In this paper we first describe the importance of theory followed by how theory driven research changes the manner researchers interact with decision-makers. We conclude on how theory driven research may influence the training, practice and the funding of health services research.

## Discussion

### The importance of theory

In recent years a number of researchers have advocated a greater role for the use of theory in strengthening the practice of research [8-12]. However, health services research has continued to focus primarily on evaluating outcomes with less attention to the mechanisms by which these outcomes are produced [10,13,14]. The emphasis on method at the expense of theory has led to several criticisms. Chen and Rossi [15] argue that an atheoretical approach to research is characterized by adherence to a step-by-step cookbook method for doing outcomes studies. In this situation they contend that research is reduced to a set of predetermined steps that are mechanically applied to various interventions without concern for the theoretical implications of intervention content, setting, participants or implementing organizations. The atheoretical approach tends to result in a simple input/output, or black box type of study [13]. Such simple evaluations may provide a gross assessment of whether or not an intervention works under one set of conditions but fail to identify the reasons why. As such, the conclusions are often less than satisfying to consumers of research results and not easily transferable to different settings.

Theory provides a systematic view of a phenomena by specifying the relations among variables and propositions with the purpose to explain or predict phenomena that occurs in the world [16,17]. In health services research theory can provide a framework to understand the relationship between program inputs (resources), program activities (how the program is implemented) and their outputs or outcomes [11,13]. In addition to identifying the mechanisms by which programs are effective, theory may consider program implementation and contextual factors. While it is important to know the extent to which an intervention attains intended outcomes, it is also essential to know what occurred in the implementation of the intervention. Variation in the implementation of the intervention may be due to differences among program providers, target population characteristics, and differences among sites on how the intervention is delivered. Theory also offers the opportunity to specify the contextual conditions that will influence the effectiveness of an intervention. Attitudinal factors at the provider level as well as structural, cultural factors at the organizational level have been under appreciated in exploring variations in health care outcomes [9,18,19]. Understanding the influence that contextual factors have on program implementation and outcomes facilitates successful application of the intervention in alternate settings, therein, addressing the generalizabilty of an intervention.

Theory offers many advantages to the health services researcher. Theory helps to identify the appropriate study question and target group; clarify methods and measurement issues; provide more detailed and informative descriptions on characteristics of the intervention and supportive implementation conditions; uncover unintended effects; assist in analysis and interpretation of results; and, the successful application of an intervention to different settings [11,12].

Theory-driven studies are addressing the challenge of both decision-makers and funding agencies to move beyond simplistic explanations of significance in health services research. Decision-makers are seeking explanations about how an intervention works and whether it will work in a fashion similar to the intervention that was evaluated when applied to a different environment [10,12,20].

Despite these potential benefits, there are a number of reasons offered as to why there has been a failure to integrate theory into research. Ironically, clinical randomized control trials have discouraged the use of theory in health services research. Given the genesis of clinical trial methodology, this may derive, in part, from the very origins of epidemiology, whereby John Snow allegedly ended an epidemic of cholera by removing the handle from the Broad Street water pump, even though he had no concept of what actually caused cholera. By ignoring the need for theory, Snow was able to overcome the fact that the theories he would have needed had not yet been elucidated. Similarly, we know that lung cancer incidence can be reduced by elimination of cigarette smoking, even though we do not know exactly how cigarette smoke causes lung cancer. Experimental trials often determine intervention effects without considering how the component features of an intervention work together to bring about study outcomes [13,15,21]. The more complex the intervention, the more difficult it is to know what the treatment entailed. There is a growing recognition for the need to establish the theoretical bases of interventions. The United Kingdom Medical Research Council recently proposed a framework for the development and evaluation of randomized control trials for complex interventions where theory is viewed as valuable in assisting hypothesis development and steering decisions on strategic design issues [22].

Adopting a theory-driven approach in health services research is not without its challenges. Given the typical training of researchers and the uni-disciplinary nature of the practice the first challenge is the capacity of researchers to engage in theory driven research. Second, a theory driven approach requires organizational conditions that support researchers and decision makers collaborating in the development and testing of theory. Finally, theory development and testing is cumulative in nature, encouraging researchers to pursue a programmatic approach in research. This approach has implications on how funding agencies support health services research. Despite the potential challenges, a theory based approach offers promise for a greater understanding on what happens when interventions work to address social/health problems.

### The importance of collaboration with decision-makers

Collaborative research partnerships between academic researchers and decision-makers describe a relationship and process between individuals from different backgrounds, who together, develop an integrative cooperative approach to resolve a research problem [23]. It has been identified as a significant strategy that holds multiple benefits [23-27].

Collaborative practice has also been identified as a key strategy in facilitating a theory driven approach. Weiss [28] recommends that the first criterion in selecting a theory to guide the evaluation of a program is to draw the theory out from those associated from the program, including designers of the program, program personnel and relevant clinical staff. The argument is that few programs are theory driven. Rather, they are typically the product of the experience and values of those who are associated with the program. In recent years a number of techniques have been developed for this purpose. Strategies range from unstructured interviews, to highly structured iterative interactions between program personnel and researchers [29-31]. Perspectives of service providers can be rounded out by a review of the research literature. In fact, a number of researchers suggest a combination of these two approaches [10,32].

Viewing program stakeholders as a key source in developing theory in health services research demands stronger collaboration between researchers and program decision makers [9]. In this fashion, collaborative practice becomes a methodological strategy in health services research. Lomas [7] has stressed that a first step in encouraging meaningful partnerships between researchers and decision-makers is to view linkage and exchange between the two as a process not as a discrete event. Establishing and maintaining ongoing links offers a more comprehensive understanding between the two groups. Researchers uncover the desired program outcomes, the causal change of the program intervention and develop a better understanding of the contextual factors that influence the variation on intervention implementation and outcomes. Similarly, decision-makers will develop a deeper understanding of the research process and thus can influence the development of feasible and sustainable interventions for practice settings.

The role and impact of the researcher and the research process in practice settings have received greater attention in other fields such as program evaluation, nursing, anthropology and community psychology. For example, core principles of community psychology practice include: a) consistency of goals and values between the researcher and the setting, and b) the notion that interventions should have the potential for being "institutionalized" or systematically established within the setting in such a way that strengthens the natural resources of the setting [25-27]. Rather than reinventing the wheel, health services research could benefit from theoretical frameworks developed within these disciplines.

### Implications for the practice of health services research

Recognizing the importance of theory calls for new expectations in the practice of health services research. There are a number of challenges that must be met in order for these perspectives to gain acceptance in the health services research community.

Evolving perspectives on the practice of health services research require recognition that few disciplines are able to span the breadth of responsibilities associated with the research process. To date there has been a tendency for health services research to be practiced as a uni-discipline where clinical disciplines tend to practice separately from the social science disciplines. A priority is to encourage the formation of research teams that are inter-disciplinary. Pursuing this agenda will promote the formation of research teams that may include: business, anthropology, sociology, psychology, education, engineering, nursing and medicine. Combined disciplinary skills would, in a complementary fashion, address the breadth of skills required in a more complex research environment that includes the development and testing of theory.

A second point concerns broadening the training for those who will practice health services research. By and large, academic training has focused on methodological issues. While a focus on research methods has made an important contribution to the practice of health services research, relying on research methods as a core curriculum has led to limitations in the training of health services researchers such as inadequate attention to the value of theory driven research. As health services research expands its methodological repertoire beyond the classical randomized control trial, researchers face increased ambiguity in attributing the source of intervention impact. It is in this circumstance that theory can guide health services researchers in understanding the causal linkages within an intervention. Further, students are educated in separate departments with little planned, formal activity across disciplines, which discourages co-operative approaches to research and service [33-35]. Education programs are not generally structured to facilitate the importance of inter-disciplinary strategies. Identifying the processes associated with creating effective linkages between researchers and decision-makers are also not typically part of training. Rethinking the current assumptions and practices regarding the training of health services researchers will enable trainees in health services research to be better prepared for their evolving responsibilities.

Collaboration between researchers and decision-makers are contingent upon supportive organizational conditions for both partners. Researchers have, and most likely will continue to operate from university-based settings where incentives for promotion and tenure can act as barriers to changes in the practice of health services research [7,36]. Most academic institutions award tenure and promote faculty based upon the frequency and quality of publications and on obtaining peer review funding [36]. The time involved in collaborating with decision-makers, joint planning and implementing research often represents activities that are not recognized by tenure promotion committees. As well, these activities may slow the production of research results and the generation of publications. Recognizing these factors requires academic centres to generate new criteria for evaluating contributions to knowledge and practice. Decision-making organizations also play a significant role in ensuring the success of collaborative relationships. The clearest indication of institutional support for research is to provide the time and resources for decision-makers to participate in collaboration activities with researchers.

Funding bodies have the potential to play a significant role in guiding and integrating these considerations into health services research. Research sponsors can develop evaluation criteria that encourage the application of theory. As an example, the Agency for Healthcare Research and Quality (AHRQ) in the USA funded an initiative (Translating Research into Practice) to identify sustainable and reproducible strategies that will: 1) accelerate the impact of health services research on direct patient care; and 2) improve the outcomes, quality, effectiveness, efficiency, and/or cost effectiveness of care through partnerships between health care organizations and researchers [37]. Further, research sponsors are beginning to move away from supporting single shot studies that are conducted in relative independence from one another. This focus on supporting programmatic research should be encouraged. Programmatic research offers a cumulative environment that allows researchers the opportunity to develop and test the application of theory. In a similar fashion, collaborative practice is also best practiced in a programmatic environment. Developing and maintaining linkages with decision-makers is predicated on developing and maintaining long-term relations. Embedded within these linkages are fundamental professional and personal attributes that include; credibility, familiarity, mutual understanding and trust [38,39].

## Summary

This paper has examined the importance of theory in health services research. We have argued that by strengthening the role of theory encourages collaborative practice between researchers and decision-makers. It has been noted that a theory driven approach in health services research is not without its challenges. However, given the modest advances towards incorporating research evidence into healthcare decisions, a theory driven approach is well worth the effort. The implication of this approach for health services research is that it has impact on the training and practice of health services research. Institutions and researchers should consider this emerging model of practice if health services research is to fulfill its potential for improving the delivery of care.

## Competing interests

The authors declare that there are no competing interests. Disclaimer: The opinions expressed are the authors' and do not necessarily represent official policy of AHRQ or the Department of Health and Human Services

## Authors' contributions

KB drafted the manuscript, edited and revised the contents, EO edited and revised the manuscript, KB, EO, MC, RS, DS all contributed to the conceptual development, editing and review of the manuscript.

## Pre-publication history

The pre-publication history for this paper can be accessed here:


